# Pyramidal and extrapyramidal dysfunction as a sequela of hypoxic injury: case report

**DOI:** 10.1186/1471-2377-7-18

**Published:** 2007-06-27

**Authors:** Martina Vendrame, S Ausim Azizi

**Affiliations:** 1 Department of Neurology, Temple University School of Medicine, Philadelphia, USA

## Abstract

**Background:**

The clinical and radiological aspects of hypoxic brain injury without ischemia are not well characterized. A spectrum of clinical manifestations have been observed in patients that recover from hypoxic brain injury, including a subset that demonstrate persistent motor system disturbances. Early Magnetic Resonance Imaging (MRI) studies have shown abnormalities in basal ganglia, cerebral and cerebellar cortex.

**Case presentation:**

A 23-year-old man was affected by acute respiratory failure after drug overdose. His clinical condition progressed from coma to partial recovery with persistent lack of control and stiffness in the lower extremities. MRI of the brain showed evolving lesions in the cerebellum, globus pallidus and motor cortex that correlated with neurological signs.

**Conclusion:**

A careful analysis of this case and a review of the relevant literature indicate that the clinical residua after recovery from hypoxic injury to the brain is predominantly disorders of the motor system, and the MRI manifestations as well as the clinical presentation can evolve over time. Understanding more of the factors that affect hypoxic brain injury can be helpful in determining the clinical outcome and management of these patients.

## Background

While it is widely accepted that hypoxic and ischemic brain injuries represent two different pathological entities, the clinical and radiological aspects of pure hypoxic brain damage are not well characterized. Depending on the severity of hypoxic insult, a wide range of clinical residues has been described. These vary from no clinical deficits [1-3] to coma and persistent vegetative states as the late sequelae [4,5]. However, a subset of young patients that recover from anoxic brain injury demonstrate persistent motor system disturbances, sometimes extending weeks to months after the initial injury [6,7]. Magnetic Resonance Imaging (MRI) can provide insights into distribution, severity and progression of hypoxic injury [8]. Similar to the pattern of injury due to ischemia, the early MR studies of patients after hypoxic brain injury have shown abnormalities in basal ganglia, cortex, hippocampus and the cerebellum [4-6].

In this report, we describe a case of anoxia without ischemia after respiratory failure secondary to drug overdose. Clinical manifestations of this case and a review of the literature indicate that the predominant clinical sequelae of pure hypoxic injury to brain are pyramidal and extrapyramidal dysfunctions, and that these symptoms can evolve over a period of weeks after the initial static injury.

## Case presentation

A 23-year-old right-handed man was found unconscious in his house about 12 hours after he was last seen in his usual state of health. On site, he was cyanotic and had a pulse oxymetry saturation of 81%. The blood pressure on the scene was 146/78 mmHg, with a heart rate of 101 and respiratory rate of 5/min. He was unresponsive to painful stimuli and had a Glasgow Coma scale of 4. He was intubated on the field and transferred to the emergency department.

**Figure 1:**
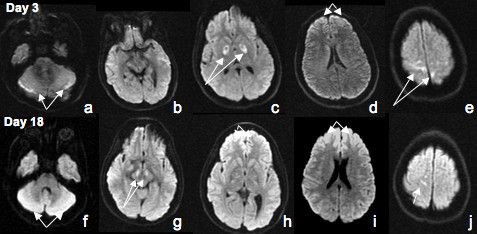
**Top row**: DWI sequences of MRI brain obtained at day #3 after admission showing abnormal signal in the cerebellum (a), globus pallidus bilaterally (c), motor cortex (d and e). **Bottom row**: DWI sequences of MRI brain at day #18 after admission showing more attenuated and confluent cerebellar lesions (f), new hyperintensities in the cerebral peduncles (g), and frontal cortex (h and i), persistent smaller lesions in the motor cortex (j).

**Figure 2:**
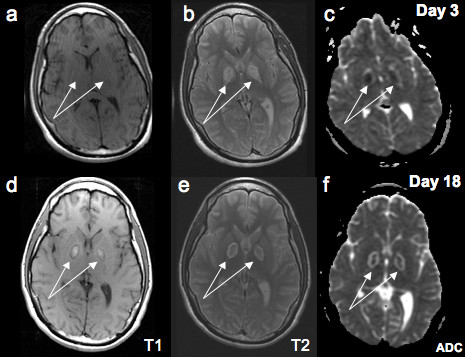
**Top row**: T1 (a), T2 (b) and ADC (c) images obtained at day #3 after admission showing abnormal signal in the globus pallidus in all sequences except T1. **Bottom row**: T1 (d), T2 (e) and ADC (f) images obtained at day #18 after admission showing persistent abnormalities in the globus pallidus that now appeared clearly in the T1 weighted sequences.

On neurological examination, he was unconscious; he had intact pupilary reflexes with positive corneal and gag reflexes. His face was symmetric. He had no spontaneous limb movements, and assumed a decerebrate posture upon noxious stimulation. Reflexes were brisk and symmetric and the plantar reflexes were extensor. Urine drug screen was positive for cocaine, opiates and cannabin metabolites. MRI of brain, obtained 2 days after the episode, showed abnormal signal in Diffusion Weighted Imaging (DWI) sequences in the cerebellum (Figure 1a), motor cortex (Figure 1e) and in the globus pallidus bilaterally (Figure 1c). The globus pallidus lesions were also evident in the apparent diffusion coefficient (ADC) sequences and T2 weighted sequences but not in the corresponding areas of T1 weighted images (Figure 2, upper panel).

In the first 48 hours, he rapidly improved and was extubated. One week after the injury, he was awake, alert and able to follow 2-step commands. His anterograde memory was intact and he recalled 2 out of 3 names in the 3 object recall test. He was able to perform simple calculations. His speech was slow and dysarthric. He could move his arms but his hand movements were clumsy, and he had difficulty in volitionally moving his legs. There was a significant increase in muscular tone, more prominent in his legs. Reflexes were brisk in the arms and were symmetric; his plantar reflexes remained extensor.

A second MR imaging of the brain was performed two weeks after injury. The cerebellar lesions that were observed in the first DWI sequences were attenuated and confluent (Figure 1f). There were new hyperintensities in the cerebral peduncles (Figure 1g), and in the frontal cortex (Figure 1h and 1i). Smaller lesions in the motor cortex were again noted (Figure 1j). Previous abnormalities that were observed in the globus pallidus were now less apparent in the DW images (Figure 1h), but appeared clearly in the T1 weighted sequences (Figure 2d). These lesions remained visible in the T2 and ADC sequences (Figure 2e and 2f).

His neurological examination improved during the following two weeks of hospitalization. His motor exam showed persistent loss of control and stiffness in the lower extremities. Upon discharge (24 days after admission), he was able to stand up but unable to ambulate normally, secondary to impaired balance, and stiffness and impaired control of his legs. Three months after discharge, he was seen for a follow up examination. His mental state and speech were within normal limits. His motor exam showed improved strength in his lower extremities but with impaired motor control and significant stiffness. He was ambulating with the aid of a walker.

## Conclusion

In this report, we evaluated the pattern of hypoxic damage across 2 imaging studies over a period of two weeks and correlated these finding with the clinical signs and symptoms up to 15 weeks after the injury. Our patient remained affected by significant loss of motor control and increased extensor tone in the lower extremities; he developed impaired balance and inability to ambulate that improved minimally. These findings are consistent with damage to the motor system including globus pallidus, cerebellum and frontal lobes. Despite the significant motor problems, the sensory and the non-executive cognitive systems of the patient remained intact. A review of the literature on the reported cases of hypoxic brain injury reveals that the predominant clinical residues after pure hypoxic injury are motor disorders, and that the motor disturbances may manifest after significant latency [7,9-11]. For example, Feve et al. [9] have described that lesions confined to globus pallidus after a hypoxic brain insult are associated with residual axial motor disturbances. Movement disorders have been described in young children years after the initial hypoxic brain injury [7].

Pure anoxic insult without ischemia does not cause brain necrosis in experimental models [12] and in humans [13]. Also, there are reports of cases of severe hypoxia that have shown a seemingly full recovery with absent neurological deficits [2,3]. It is likely that brain cell death secondary to hypoxia occurs more by apoptosis, and as such, the clinical and imaging findings take several weeks or perhaps longer to mature [8,14]. Diffusion weighted MR imaging is a sensitive tool that can detect alterations in diffusion of protons within the brain tissues, both gray and white matters, and it is commonly used to evaluate hypoxic injury in early stages. It appears that the cell layers within the neocortex, hippocampus, cerebellum, putamen and caudate are the most affected areas [15,16]. In our patient, after 2 weeks, in addition to the initial hyperintense signals in the globus pallidus, confluent hyperintensities in the cerebellum and new lesions in the frontal lobes and cerebral peduncles became apparent (Figure 1, lower panel), indicating that despite the static nature of the insult the damage and recovery in the brain tissues evolved. Selective vulnerability of different brain areas such as the basal ganglia to a diffuse insult may be related to differential neuronal activity, resulting in regional variations in cell death. In addition, the locations and topography of lesions suggest that it is unlikely that an ischemic/hypovolemic event contributed to the observed damages, as lesions secondary to hemodynamic failure are often restricted to the "watershed" territories [17].

Another striking feature is bilateral lesions within the globus pallidus. They initially appeared in DW sequences, became attenuated in subsequent DW sequences but became more prominent in T1 weighted images. Similar abnormalities have been shown after carbon monoxide (CO) [6,18] and methanol [19,20] intoxication. Poisoning with these substances causes tissue hypoxia, also known as histotoxic hypoxia [13]. Our MRI findings are actually similar to these anoxic injuries to the brain. Similar lesion topography have been seen after heroin intoxication when injected in combination with cocaine [21] or alone [22]. However, in these cases the most probable mechanism for heroin-induced ischemia was thought to be hypoxia (likely secondary to decrease respiratory drive) [22]. It is also relevant to consider that other drugs may contribute to the pattern and severity of brain damage secondary to hypoxia. Our patient drug screening was positive for cocaine, opiates and cannabis. Cerebral infarctions associated with cocaine use have been described [23]. But, the damage is usually lobar and only rarely affects the basal ganglia symmetrically [20,21]. It is known that cocaine changes the neuronal activity in the basal ganglia [24]. Therefore, the pattern of hypoxia-induced damage to the basal ganglia and indeed other areas of the brain can be dictated by the pattern of neuronal activity in the nervous system.

The MRI findings observed in our case are congruent with the clinical manifestations of the hypoxic insult; and similar to other reports, the clinical sequelae are predominantly motor disorders. The symptoms evolved over time in parallel with imaging findings. It appears that hypoxic injury to the brain is a dynamic process and understanding all the factors contributing to this process is fundamental in determining the significance of initial clinical and radiological evaluation for managing and predicting functional outcomes of these patients.

## Competing interests

The author(s) declare that they have no competing interests.

## Authors' contributions

MV reviewed the existing literature and drafted the manuscript which was edited by SAA. Both authors reviewed and selected radiology images. Both authors read and approved the final manuscript.

## Pre-publication history

The pre-publication history for this paper can be accessed here:



## References

[B1] Jason GW, Pajurkova EM, Lee RG (1989). High-altitude mountaineering and brain function: neuropsychological testing of members of a Mount Everest expedition. Aviat Space Environ Med.

[B2] Sadove MS, Yon MK, Hollinger PH, Johnston KS, Phillips FL (1961). Severe prolonged cerebral hypoxic episode with complete recovery. Jama.

[B3] Gray FD, Horner GJ (1970). Survival following extreme hypoxemia. Jama.

[B4] Els T, Kassubek J, Kubalek R, Klisch J (2004). Diffusion-weighted MRI during early global cerebral hypoxia: a predictor for clinical outcome?. Acta Neurol Scand.

[B5] Hald JK, Brunberg JA, Dublin AB, Wootton-Gorges SL (2003). Delayed diffusion-weighted MR abnormality in a patient with an extensive acute cerebral hypoxic injury. Acta Radiol.

[B6] Singhal AB, Topcuoglu MA, Koroshetz WJ (2002). Diffusion MRI in three types of anoxic encephalopathy. J Neurol Sci.

[B7] Scott BL, Jankovic J (1996). Delayed-onset progressive movement disorders after static brain lesions. Neurology.

[B8] Sagar P, Grant PE (2006). Diffusion-weighted MR imaging: pediatric clinical applications. Neuroimaging Clin N Am.

[B9] Feve AP, Fenelon G, Wallays C, Remy P, Guillard A (1993). Axial motor disturbances after hypoxic lesions of the globus pallidus. Mov Disord.

[B10] Kuoppamaki M, Bhatia KP, Quinn N (2002). Progressive delayed-onset dystonia after cerebral anoxic insult in adults. Mov Disord.

[B11] Wallays C, Feve A, Boudghene F, Fenelon G, Guillard A, Bigot JM (1995). [Hypoxic cerebral lesions. X-ray computed tomography and MRI aspects. Apropos of 20 cases. Selective vulnerability of the striatopallidum]. J Neuroradiol.

[B12] de Courten-Myers GM, Yamaguchi S, Wagner KR, Ting P, Myers RE (1985). Brain injury from marked hypoxia in cats: role of hypotension and hyperglycemia. Stroke; a journal of cerebral circulation.

[B13] Miyamoto O, Auer RN (2000). Hypoxia, hyperoxia, ischemia, and brain necrosis. Neurology.

[B14] Grant PE, Yu D (2006). Acute injury to the immature brain with hypoxia with or without hypoperfusion. Radiologic clinics of North America.

[B15] Arbelaez A, Castillo M, Mukherji SK (1999). Diffusion-weighted MR imaging of global cerebral anoxia. AJNR Am J Neuroradiol.

[B16] Chalela JA, Wolf RL, Maldjian JA, Kasner SE (2001). MRI identification of early white matter injury in anoxic-ischemic encephalopathy. Neurology.

[B17] Bladin CF, Chambers BR (1994). Frequency and pathogenesis of hemodynamic stroke. Stroke; a journal of cerebral circulation.

[B18] Prockop LD (2005). Carbon monoxide brain toxicity: clinical, magnetic resonance imaging, magnetic resonance spectroscopy, and neuropsychological effects in 9 people. J Neuroimaging.

[B19] Sefidbakht S, Rasekhi AR, Kamali K, Borhani Haghighi A, Salooti A, Meshksar A, Abbasi HR, Moghadami M, Nabavizadeh SA (2007). Methanol poisoning: acute MR and CT findings in nine patients. Neuroradiology.

[B20] Hantson P, Duprez T (2006). The value of morphological neuroimaging after acute exposure to toxic substances. Toxicological reviews.

[B21] Daras MD, Orrego JJ, Akfirat GL, Samkoff LM, Koppel BS (2001). Bilateral symmetrical basal ganglia infarction after intravenous use of cocaine and heroin. Clinical imaging.

[B22] Vila N, Chamorro A (1997). Ballistic movements due to ischemic infarcts after intravenous heroin overdose: report of two cases. Clinical neurology and neurosurgery.

[B23] Jacobs IG, Roszler MH, Kelly JK, Klein MA, Kling GA (1989). Cocaine abuse: neurovascular complications. Radiology.

[B24] Rebec GV (2006). Behavioral electrophysiology of psychostimulants. Neuropsychopharmacology.

